# Apocrine lesions of the breast

**DOI:** 10.1007/s00428-021-03185-4

**Published:** 2021-09-18

**Authors:** Cecily M. Quinn, Clare D’Arcy, Clive Wells

**Affiliations:** 1 Irish National Breast Screening Programme and Department of Histopathology, St. Vincent’s University Hospital, Elm Park, Dublin 4, Ireland; 2 School of Medicine, University College Dublin, Dublin, Ireland; 3 University College Hospital, London, UK

**Keywords:** Breast, Apocrine, Atypia, In situ carcinoma, Carcinoma with apocrine differentiation, Molecular apocrine tumour

## Abstract

Apocrine change is recognised in benign, atypical and malignant lesions of the breast. Apocrine metaplasia, a frequent finding in the breast of women over the age of 25 years, is most commonly seen in benign cysts with a simple or papillary configuration. Apocrine change is also recognised in other benign lesions including sclerosing adenosis, now known as apocrine adenosis. Apocrine atypia usually refers to cytological atypia in which there is at least threefold variation in nuclear size but architectural atypia may also occur. The distinction between atypical apocrine hyperplasia and non-high-grade apocrine ductal carcinoma in situ may be difficult due to the relative rarity of these entities and the lack of validated diagnostic criteria. Lobular carcinoma in situ (LCIS) with apocrine change is considered to be a variant of pleomorphic LCIS. An apocrine variant of encapsulated papillary carcinoma is also recognised. Apocrine change is described in invasive carcinoma, including no special type, lobular, micropapillary and mucinous variants. The recent WHO 2019 update recognises ‘carcinoma with apocrine differentiation’ as a special type breast carcinoma based on the presence of apocrine morphology in at least 90% of the tumour. Tumours with apocrine morphology are usually but not always hormone receptor negative. Human epidermal growth factor receptor 2 (HER-2) status is variable. Molecular studies have identified breast tumours with apocrine features and high expression of androgen receptor mRNA including ‘luminal androgen receptor tumours’ and ‘molecular apocrine tumours’. The term ‘pure apocrine carcinoma’ has been proposed to describe an invasive carcinoma with apocrine morphology that is oestrogen and progesterone receptor negative and androgen receptor positive. HER-2 status may be positive or negative. This article reviews the pathology of benign, atypical and malignant apocrine lesions of the breast, with emphasis on diagnostic criteria including an approach to evaluation of apocrine lesions on needle core biopsy, and recent advances in our understanding of invasive apocrine carcinoma.

## Introduction

Apocrine cells are extremely common in the breast and are considered to represent a metaplastic phenomenon [1]. Apocrine cells usually assume a cuboidal or columnar appearance with frequent apical blebs or snouts. Two morphological variants are recognised. Type A cells are characterised by dense, eosinophilic cytoplasm, frequently incorporating a supra-nuclear vacuole that is rich in haemosiderin. Type B cells have foamy cytoplasm with multiple vacuoles that may coalesce. Both cell types have round nuclei that may display considerable variation in size and frequently contain prominent nucleoli. These latter features, together with the architectural complexity frequently seen in apocrine change, may lead to challenges in the precise categorization of apocrine breast lesions.

Normal apocrine cells express low molecular weight cytokeratins (CKs) 8 and 18, epithelial membrane antigen (EMA), androgen receptor (AR) [2] and gross cystic disease fluid protein (GCDFP)—15, 24 and 44 [3]. The latter are prolactin-induced protein products of the AR target gene and are frequently used as a marker for apocrine differentiation in benign and malignant lesions. Apocrine cells do not express oestrogen (ER) or progesterone (PR) receptors and are usually human epidermal growth factor receptor 2 (HER-2) negative. There is, however, a suggestion of cells intermediate between apocrine differentiation and columnar cell change in some lesions [4].

## Benign apocrine lesions

### Apocrine metaplasia in cystic change

Cyst formation is frequent in women of reproductive age and is the most common benign change in which apocrine metaplasia is observed. Cysts may be single, multiple or co-exist with other benign change, the latter commonly referred to as fibrocystic change. Women may present with symptoms or through mammographic screening. Two types of cysts have been described which differ in terms of the composition of their luminal content. Apocrine cells are present in both types and sub-categorisation according to luminal content does not appear to carry any biological significance [5]. Some studies have, however, demonstrated increased androgen and epidermal growth factor content in cysts with a high sodium to potassium ratio. This could potentially lead to a feedback loop driving apocrine cell growth via the AR [6].

**Fig. 1:**
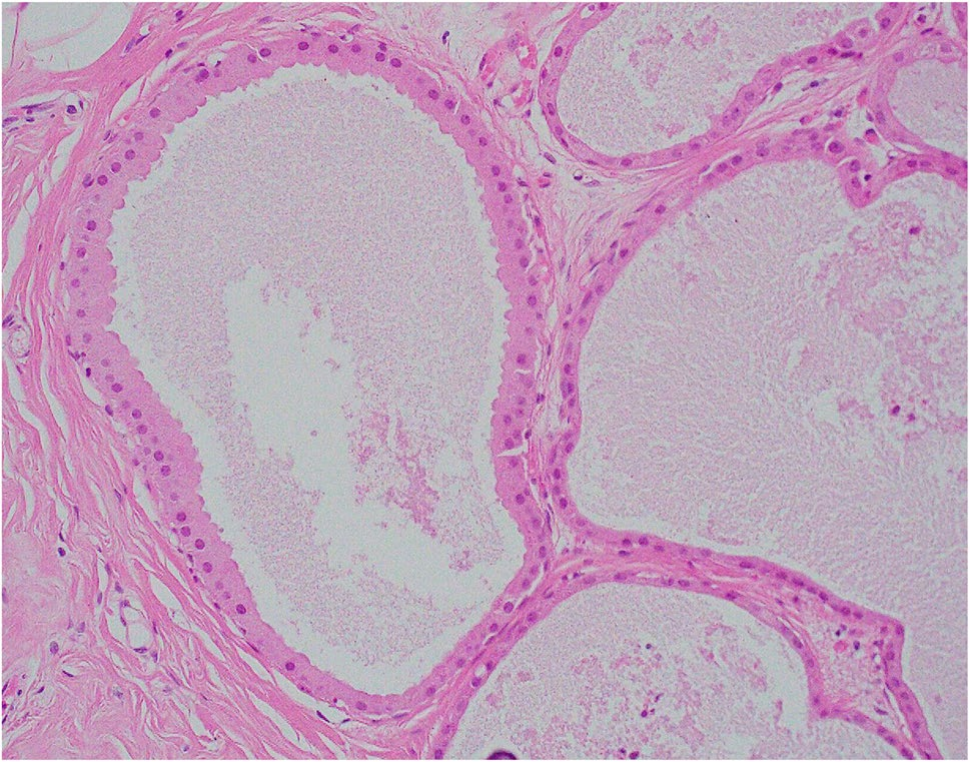
Benign cyst lined by a single, flat, layer of apocrine epithelium

**Fig. 2:**
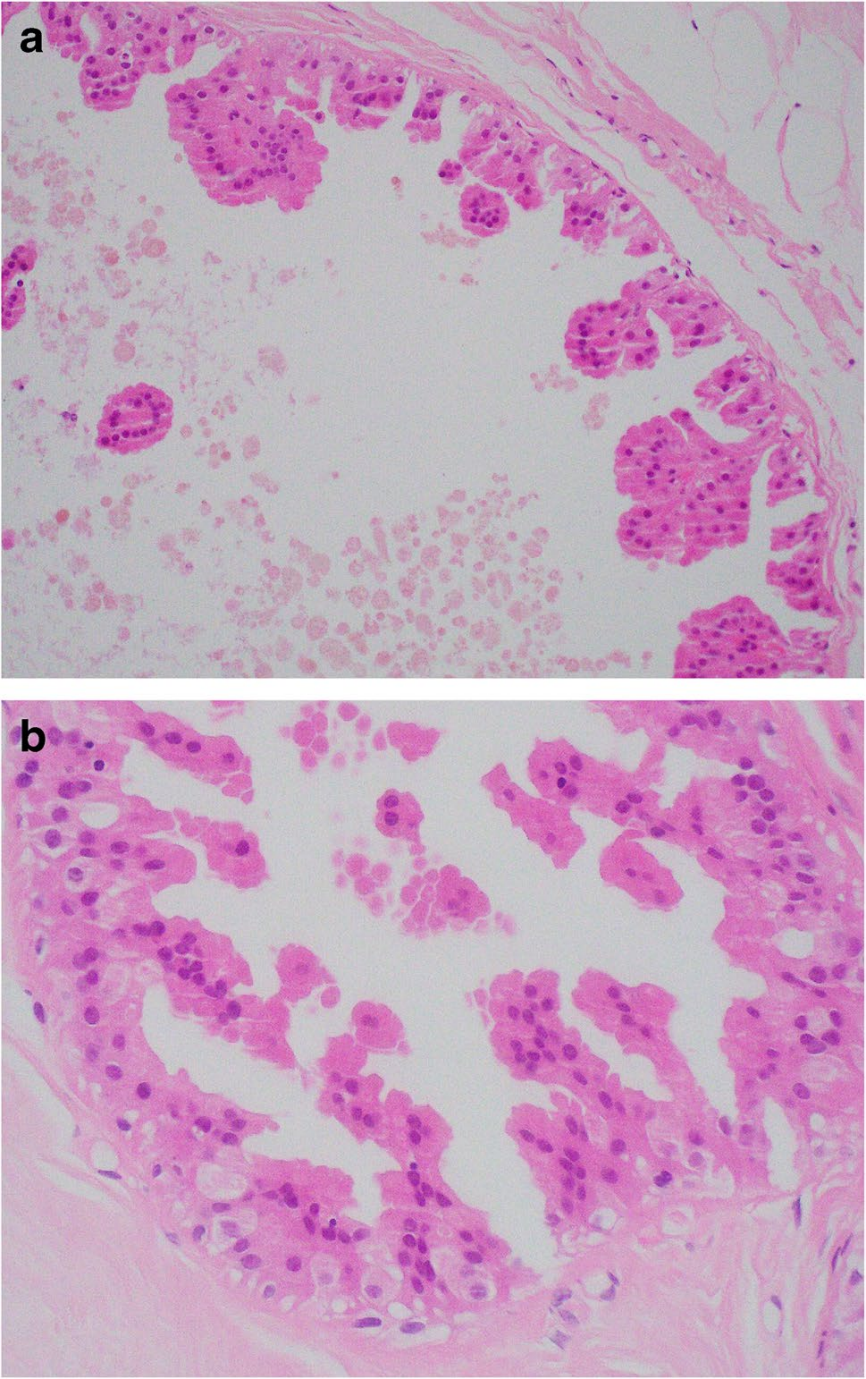
**a** Simple papillary apocrine change in which the apocrine cells are 3 or more cells thick focally and form mounds of cells, broader at the base than at the tip with no anastomoses. **b** Complex papillary apocrine change in which are taller and broader than those seen in simple papillary apocrine change with a tendency to anastomose within the lumen

Cysts may be lined by a single layer of flat, non-proliferative, apocrine epithelium (Fig. 1) with no evidence of an association with subsequent development of malignancy [7]. Apocrine epithelium lining cysts may also show varying degrees of micropapilla and papilla formation, known as papillary apocrine change. In a large series, with extensive follow-up, Page et al. classified papillary apocrine change as simple (apocrine lining cells are at least 3 or more cells thick focally resulting in ‘mounds’ of cells, broader at the base than at the tip, that do not touch each other), complex (papillae both taller and broader at the base than the mounds of simple hyperplasia with a tendency to anastomose within the lumen) and highly complex (greatly elongated papillae, 2–3 cells wide, with frequent anastomoses) (Fig. 2) [8]. Risk of subsequent malignancy was elevated but appeared to be largely due to the presence of concurrent atypia in women with simple and complex papillary apocrine change. A higher risk of subsequent malignancy was observed in women with highly complex papillary apocrine change, independent of the presence of atypia, but this did not reach statistical significance due to the rarity of this finding. The authors suggest that, although there appears to be no biological basis for distinguishing these variants of papillary change, recognition of the highly complex variant is important to avoid misinterpretation as atypical or in situ change. Variation in nuclear size may be seen in flat and papillary apocrine epithelium and should be less than three-fold.

CK 5/6 immunohistochemistry (IHC), often used to distinguish non-apocrine atypical ductal hyperplasia (ADH) (where cells lose expression) from usual epithelial hyperplasia (where partial expression is retained) is typically negative in both benign and atypical apocrine proliferations and is not useful in this context. A combination of CK5/6 and ER IHC may assist the distinction of apocrine metaplasia (ER and CK5/6 negative) from ADH (ER positive, CK5/6 negative) in certain scenarios.

### Apocrine change in sclerosing adenosis (apocrine adenosis), radial scar and papilloma

**Fig. 3:**
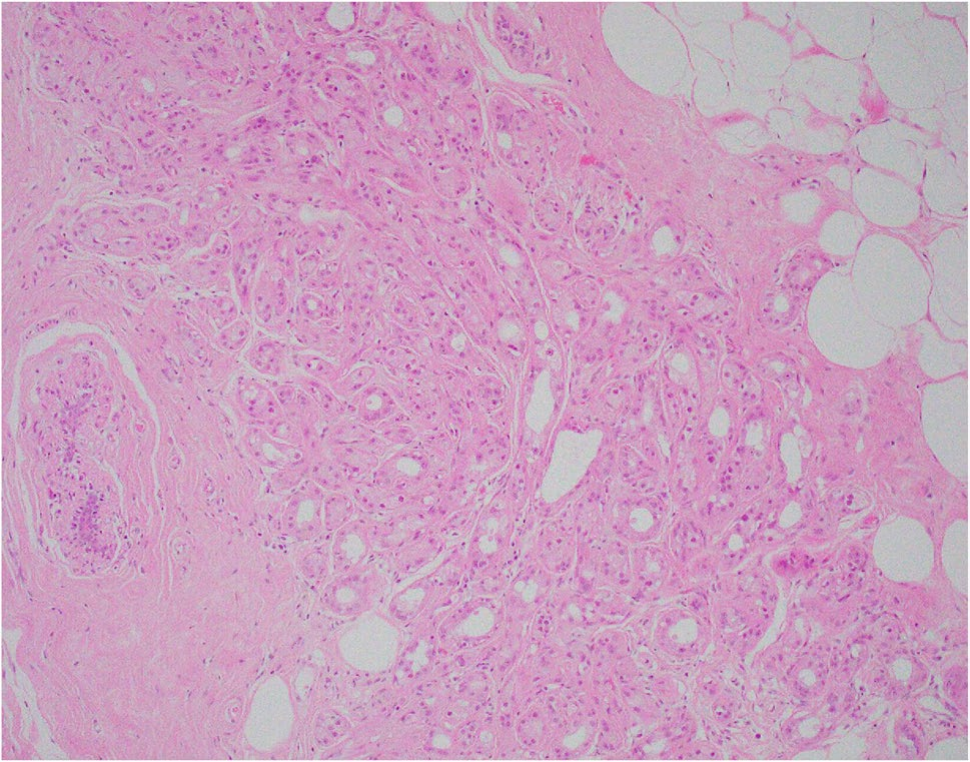
Apocrine adenosis (sclerosing adenosis with apocrine change)

Apocrine change may be seen in sclerosing adenosis, radial scars and papillomas. The term ‘apocrine adenosis’ has been variously used as a collective term to indicate apocrine change in these lesions [9], to refer only to apocrine change in sclerosing adenosis [10] and to describe a variant of adenomyoepithelioma [11]. In practice, reporting the specific entity with a comment on the presence of co-existent apocrine change, e.g. intraduct papilloma with apocrine change, avoids confusion and facilitates radiology pathology correlation [12, 13]. Currently, the term ‘apocrine adenosis’ is used to describe sclerosing adenosis with apocrine change. Variation in nuclear size should be less than three-fold (Fig. 3). Florid apocrine adenosis may be mistaken for invasive carcinoma, particularly on needle core biopsy (NCB). Myoepithelial cell markers are useful in arriving at the correct diagnosis [14]. Awareness that the myoepithelial cell layer may be attenuated or partly lost in benign apocrine lesions is important to avoid over diagnosis of malignancy [15, 16].

### Apocrine change in fibroadenomas and hamartomas

Fibroadenomas that feature cysts > 3 mm, sclerosing adenosis, epithelial hyperplasia or papillary apocrine change, termed complex fibroadenomas, have been reported to be associated with an increased incidence of malignancy [17]. However, the results of that study, reported in 1994, have not yet been confirmed by further studies. Papillary apocrine change is seen in approximately 10% of fibroadenomas and is rarely atypical. The presence of papillary apocrine change and other benign changes in fibroadenomas likely reflects the hamartomatous rather than neoplastic nature of some of these lesions. Apocrine change may be seen in hamartomas but is not seen in phyllodes tumours due to their clonal nature. In some cases, the presence of apocrine change in a benign fibro-epithelial lesion may assist the distinction of fibroadenoma from benign phyllodes tumour.

## Atypical apocrine lesions

The term apocrine atypia usually refers to cytological atypia in apocrine cells although architectural atypia may also be observed. Apocrine atypia is characterised by at least a threefold variation in nuclear size with hyperchromasia and prominent nucleoli that may be multiple [14, 18, 19], Apocrine atypia may be seen in otherwise benign sclerosing lesions including radial scar, sclerosing papilloma and sclerosing adenosis.

### Atypical apocrine adenosis

**Fig. 4:**
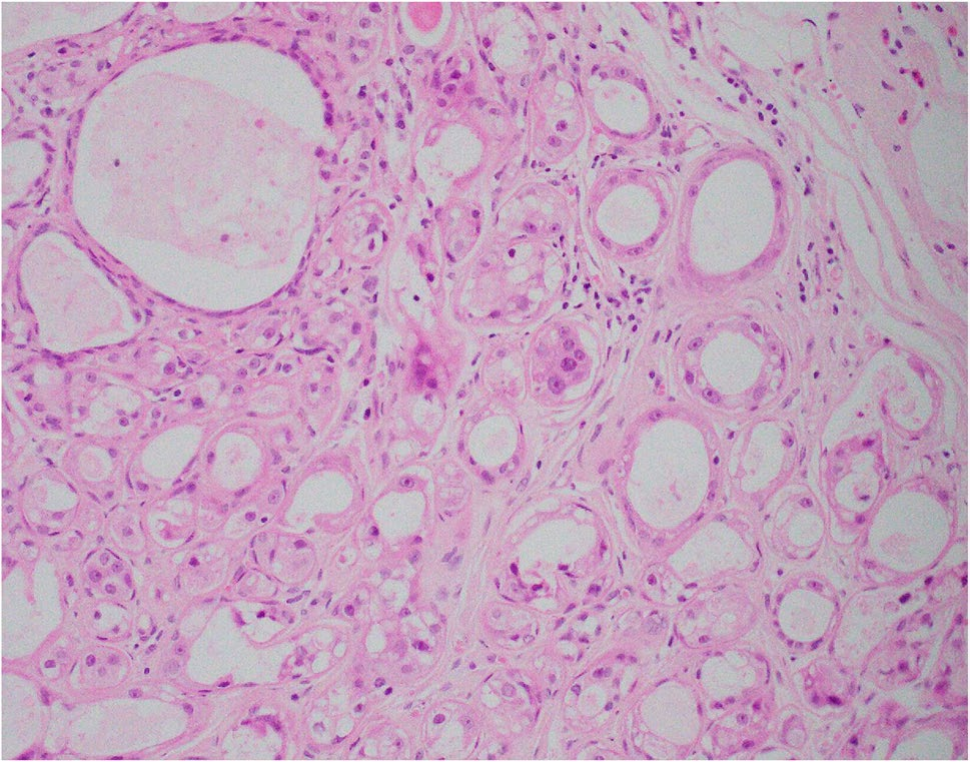
Atypical apocrine change in which there is at least threefold variation in nuclear size of the component apocrine cells

Apocrine adenosis (sclerosing adenosis with apocrine change) with superimposed atypia is referred to as atypical apocrine adenosis. Diagnosis is based on the recognition of at least a threefold variation in nuclear size (Fig. 4) [14]. Apoptosis and very occasional mitotic activity may be present. Architectural atypia and necrosis are not features of this entity. Atypical apocrine adenosis may be mistaken for invasive carcinoma on haematoxylin and eosin sections. Recognition of the lobulo-centric configuration at low power and the use of myoepithelial cell IHC will assist correct diagnosis. The myoepithelial cell layer may be attenuated and difficult to visualise so it is recommended that both nuclear (e.g. p63) and cytoplasmic (e.g. smooth muscle myosin, calponin) markers are utilised to ensure appreciation of attenuated myoepithelial cells.

The significance of atypical apocrine adenosis is uncertain due to the relative rarity of these lesions and limited clinical data. Follow-up studies that focused on outcome following excision have reported varying risk estimates [18–20]. In an upgrade study, Calhoun et al. found no cases of DCIS or invasive carcinoma in 34 diagnostic excision specimens following an NCB diagnosis of atypical apocrine adenosis when all cases with a more significant lesion on NCB were excluded [21].

### Atypical apocrine metaplasia and atypical apocrine hyperplasia

**Fig. 5:**
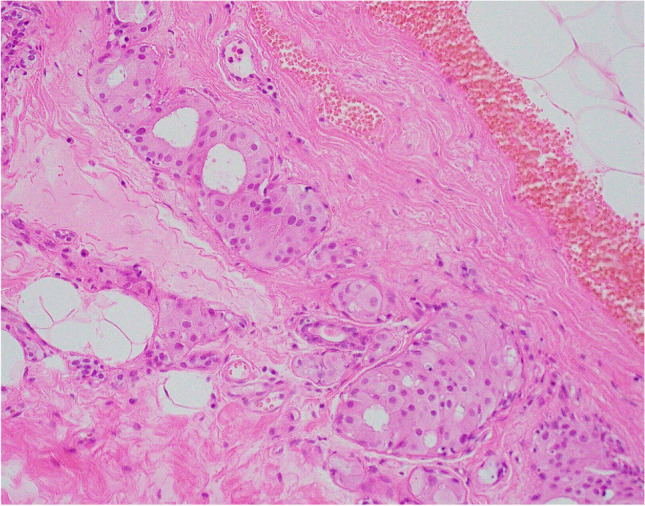
Atypical apocrine hyperplasia in which the atypia is mainly architectural due to a proliferation of apocrine cells with a solid and cribriform architecture

The term atypical apocrine metaplasia has been proposed to describe cytological atypia (at least a threefold variation in nuclear size) in apocrine epithelium lining otherwise normal or cystically dilated breast acinar structures [22]. Atypical apocrine hyperplasia (AAH) is defined as a proliferation of apocrine cells within a duct space or terminal duct lobular unit with atypical cytological features and/or architectural atypia beyond that seen in papillary apocrine hyperplasia but insufficient for a diagnosis of DCIS (Fig. 5) [1]. The presence of necrosis, abnormal mitotic activity and peri-ductal changes are not considered to be features of AAH and are more likely to indicate non-high apocrine DCIS (ADCIS) [22]. HER-2 IHC may assist the stratification of atypical apocrine proliferations. Weak membranous staining may be seen in apocrine atypia but strong expression appears to be restricted to malignant apocrine epithelium [1]. Ki-67 and p53 studies may also assist the differential diagnosis of intra-duct apocrine proliferations [23]*.* IHC, otherwise, is of limited value in distinguishing apocrine atypia from ADCIS and these lesions pose challenges in diagnostic clinical practice with inter-observer variation in categorization. Data on the clinical significance of AAH are scarce and difficult to evaluate due to varying definitions. A recent study of 17 atypical apocrine lesions diagnosed on NCB (13 AAH, 3 atypical apocrine adenosis and one combined lesion) recorded a 25% upgrade to malignancy on excision [24].

## Malignant apocrine lesions

### Apocrine DCIS

**Fig. 6:**
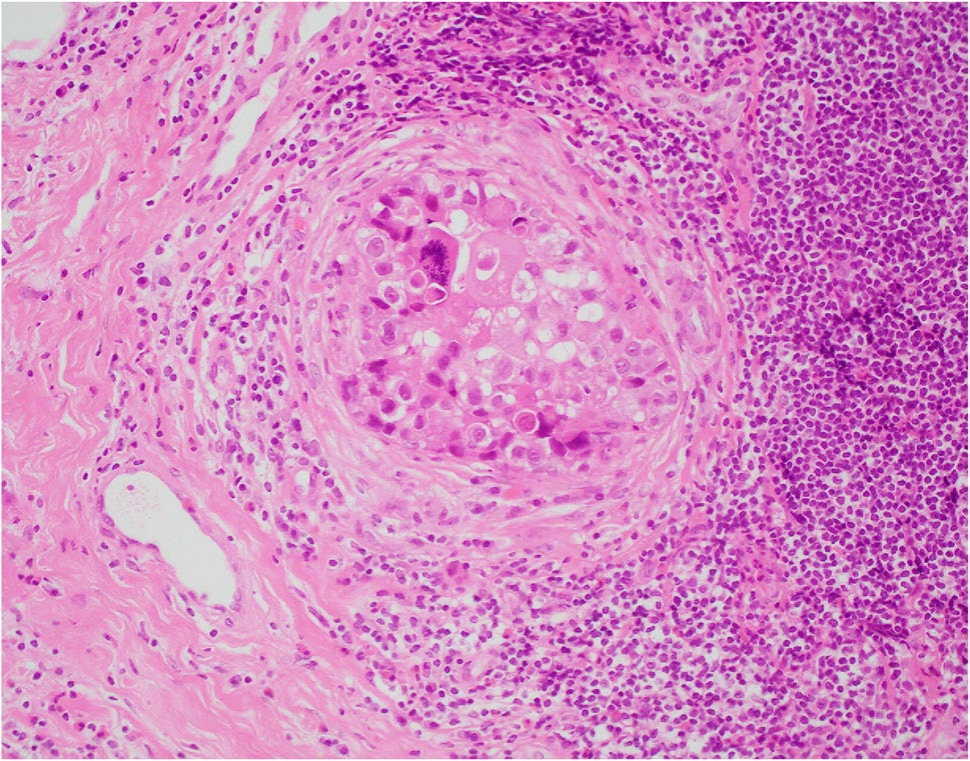
High-grade apocrine ductal carcinoma in situ (DCIS). The cells showed marked nuclear pleomorphism and abnormal mitoses. In this example, there is also dense peri-ductal chronic inflammation

Apocrine DCIS (ADCIS) is recognised as a variant of DCIS. As for other forms of DCIS, the nuclear grade may be low, intermediate or high and ADCIS shows a similar range of architectural patterns. Luminal necrosis, calcification and peri-ductal changes may be present and are more common in high grade ADCIS (Fig. 6). The latter is usually easy to recognise and diagnose due to the presence of these features in addition to marked nuclear pleomorphism and abnormal mitotic activity.

Non-high-grade ADCIS presents a more subtle morphological appearance. Various criteria have been proposed to distinguish ADCIS from AAH. Utilising cytonuclear atypia and an extent criterion of 4 mm, O’Malley et al. classified 54 atypical apocrine proliferations into DCIS, limited DCIS, borderline DCIS and atypia [13]. This approach may be useful but has not been validated in follow-up studies. Tavassoli et al. advocated the use of a size criterion of > 2 mm, as for conventional DCIS, in combination with qualitative features including cytonuclear atypia and the fully developed architectural features of DCIS in affected structures [22]. Although clinical follow-up is limited and the true biological significance uncertain, the latter approach aligns with the current criteria used to distinguish DCIS from ADH and, on the available evidence, appears to be the most pragmatic.

**Fig. 7:**
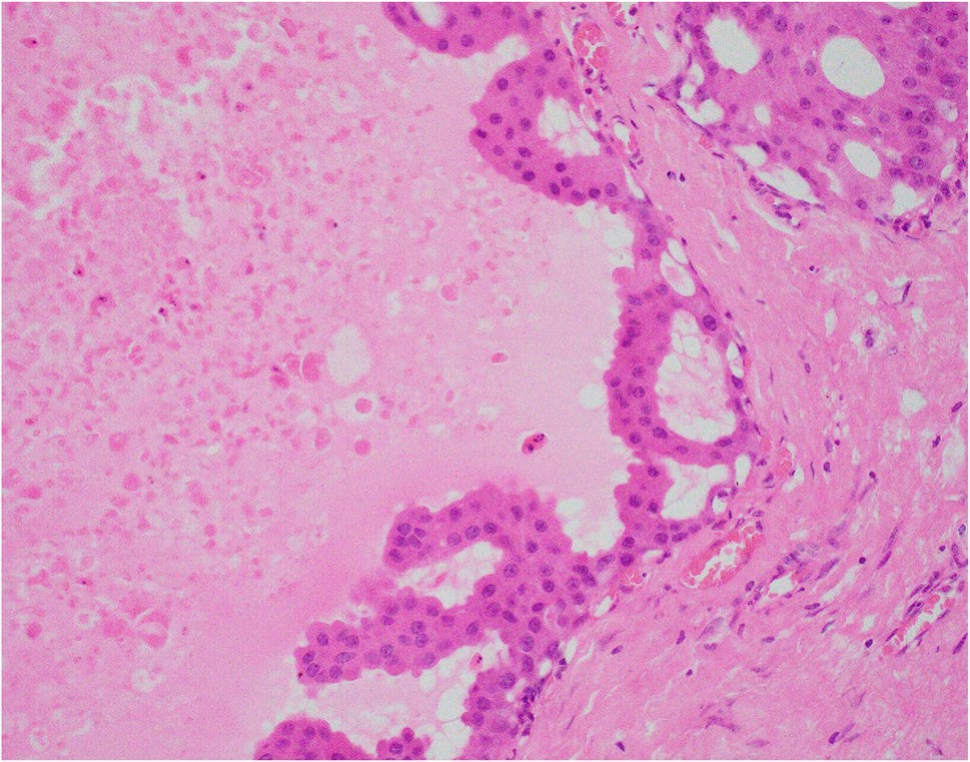
Non-high-grade apocrine DCIS showing mild cytological atypia (at least threefold variation in nuclear size) and architectural complexity. In this example, there is early necrosis in the lumen which assists the diagnosis

In summary, a diagnosis of non-high-grade ADCIS (Fig. 7) should be considered if there is duct/acinar involvement by a proliferation of apocrine cells showing cytonuclear atypia with at least threefold variation in nuclear size, fully developed architectural features of DCIS (solid, cribriform or micropapillary) and > 2 mm in extent. Mitotic activity and necrosis may not be seen. Periductal inflammation and fibrosis may be present and, in the correct context, may assist identification as ADCIS [21]. An atypical intraduct apocrine proliferation that does not fulfil the qualitative and quantitative criteria outlined above is more appropriately categorised as AAH.

There is no universally agreed method of grading for ADCIS. Using a combination of nuclear grade and the presence or absence of necrosis, Leal et al. classified 35 cases of ADCIS into low, intermediate and high grade and reported a significant correlation between high grade and Ki67 proliferative index, HER-2 expression and the presence of accompanying invasive carcinoma [25].

In practice, the diagnosis of high grade ADCIS is straightforward due to well-developed cytological and architectural features, usually with necrosis, calcification and peri-ductal changes. Recognition and accurate diagnosis of non-high-grade ADCIS is more problematic and its distinction from AAH is likely to be more clinically relevant than sub-classification into low- and intermediate-grade categories. ADCIS is usually AR positive and ER and PR negative. HER-2 expression has been reported in up to 47% of ADCIS with a higher rate of positivity in high grade lesions [25].

### *Apocrine lobular carcinoma *in situ

Apocrine morphology in lobular carcinoma in situ (LCIS) was first described by Eusebi et al. [26]. These authors reported one case with abundant foamy granular cytoplasm described as ‘histiocytoid LCIS’ and a second case with ‘abundant granular, intensely eosinophilic cytoplasm and vesicular nuclei with prominent nucleoli’. Both cases expressed GCDFP-15, confirming apocrine differentiation. Using modern criteria, these cases would be classified as pleomorphic LCIS (pLCIS) [27]. A recently reported single case of GCDFP-15-positive pleomorphic LCIS with accompanying invasive apocrine carcinoma led Ishii et al. to postulate a pathogenetic link between the two entities, both of which were triple negative [28]. In a series of 13 cases of apocrine pLCIS reported by Chen et al., the majority of patients were postmenopausal [29]. Apocrine pLCIS was defined as ‘a solid intraductal proliferation of dyscohesive cells with loss of E-cadherin expression, intracytoplasmic vacuoles, abundant eosinophilic cytoplasm and moderate to marked nuclear pleomorphism’. All cases expressed GCDFP-15, 10/13 (80%) were ER and PR negative and 4 (31%) demonstrated HER-2 amplification. Using comparative genomic hybridization analysis, apocrine pLCIS displayed a higher degree of genomic instability compared with conventional pLCIS and classical LCIS including amplification of 17q11.2 (HER-2), amplification of 11q13.3 (CCND1), 16p gain and losses at 3q, 11q, 13q and 17p. Shin et al. reported similar findings [30]. In the largest series reported to date, Zhong et al. studied 34 cases of apocrine pLCIS, including 23 with associated invasive lobular carcinoma, to fully characterise it [31]. In this study, apocrine pLCIS was defined morphologically as ‘distension of terminal duct lobular units by pleomorphic cells, with abundant eosinophilic and occasionally granular cytoplasm, intercellular dyscohesion, necrosis and calcification’. Each case was AR positive and E-cadherin negative. GCDFP-15 studies were not performed. Most cases had accompanying LCIS of lower grade. Apart from increased expression of aurora kinase A in apocrine pLCIS, there were no significant clinico-pathological differences between apocrine and non-apocrine pLCIS and no differences were detected in pure apocrine pLCIS compared with cases with accompanying invasion. Next-generation sequencing, utilising the Oncomine Comprehensive Panel v2, showed no molecular findings specific to apocrine pLCIS. In contrast to the findings of others, Zhong et al. observed that 68% of cases were ER positive and 35% PR positive. The HER-2 positivity rate was similar [31].

**Fig. 8:**
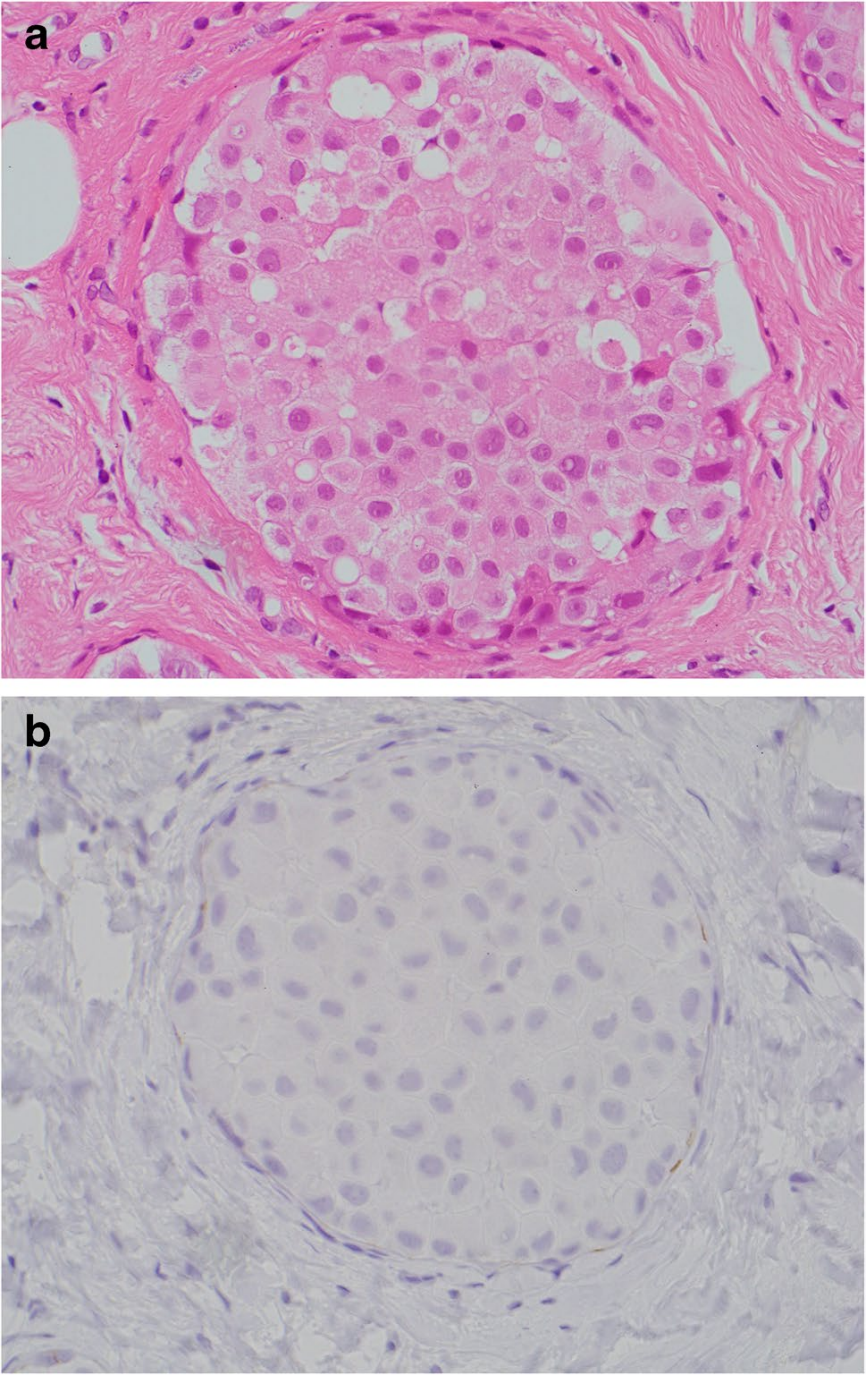
Lobular carcinoma in situ (LCIS) with apocrine morphology **a** H&E and **b** negative e-cadherin immunohistochemistry

Apocrine pLCIS may be morphologically similar to high grade ADCIS. Helpful features in the diagnosis of apocrine pLCIS include the solid cellular arrangement, dyscohesion, the presence of co-existent classical or florid LCIS and absence of e-cadherin membrane staining on IHC (Fig. 8).

Data on the natural history of pLCIS and apocrine pLCIS are limited. Studies of non-classical LCIS have reported up to 50% finding of a more significant lesion on excision [32, 33]. Recurrence rates following local excision range from 0 to 57% [34–37]. Zhong et al. observed recurrence rates of 18% and 28% in apocrine pLCIS and pLCIS respectively [31]. Although the impact of positive margin status on the likelihood of recurrence is not yet clear, the results of these studies support surgical excision to achieve clear margins and, at the present time, the WHO recommends that margin status be recorded for all cases of florid and pLCIS [27]. The biological behaviour of the apocrine variant appears to be similar to pLCIS and the results of studies to date suggest that it should be managed according to pLCIS and DCIS protocols [31, 34].

### Encapsulated apocrine papillary carcinoma

**Fig. 9:**
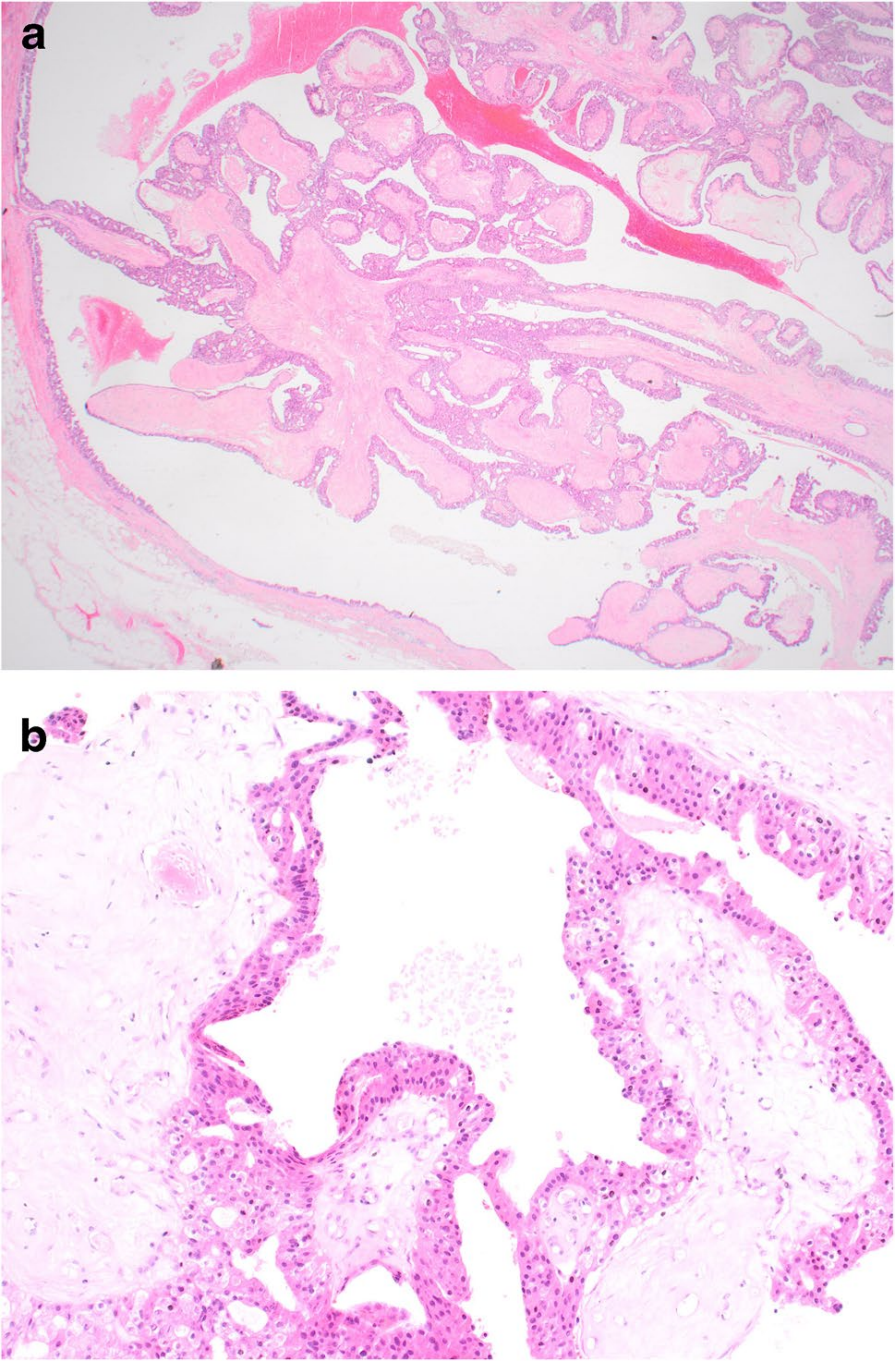
Encapsulated papillary carcinoma with apocrine change in the epithelial cell component (**a**, **b**)

Encapsulated papillary carcinoma (EPC) is a well-circumscribed, usually cystic, lesion characterised by a proliferation of anastomosing slender fibrovascular connective tissue cores lined by epithelial cells that show cytological atypia, mitotic activity and multilayering with architectural complexity, surrounded by a fibrous pseudo-capsule (Fig. 9). Both the pseudo-capsule and the fibrovascular cores are typically devoid of a myoepithelial cell lining. While these tumours are considered to represent low-grade invasive carcinoma with an expansile edge, current recommendations are to stage as in situ due to their indolent biological behaviour [38].

EPCs are typically composed of neoplastic epithelial cells with a luminal phenotype. In 2009, Seal et al. reported five cases of otherwise classical EPC in which the epithelial cell component showed apocrine morphology with cytonuclear atypia and GCDFP-15 positivity, designated apocrine EPC [39]. No myoepithelial cells were identified within or at the periphery of the lesions. Biomarker studies, performed in three cases, demonstrated a triple negative profile. Sentinel lymph node biopsy, also performed in three patients, was negative [39]. Further single case studies have observed similar morphological and IHC findings including AR positivity. Kovari et al. recently reported the first case of apocrine EPC with accompanying invasive carcinoma, also showing apocrine morphology [40]. Where clinical follow-up is available, all patients with reported apocrine EPC remain well with no evidence of recurrence. Although data are limited, the clinical behaviour of apocrine EPC appears to be similar to that of classical EPC. The single case report of co-existent invasive carcinoma highlights the importance of thorough sampling for histological evaluation.

The main differential diagnosis is benign papillary apocrine hyperplasia within a large cyst with diminished or absent myoepithelium [15]. In contrast to the latter, apocrine EPC is invested by a fibrous pseudo-capsule and should display at least mild cytological apocrine atypia. It is advisable to perform more than one myoepithelial IHC preparation (a p63 nuclear stain combined with at least one cytoplasmic stain such as calponin or smooth muscle heavy chain myosin) when searching for a myoepithelial cell layer in this setting.

### Apocrine carcinoma

**Fig. 10:**
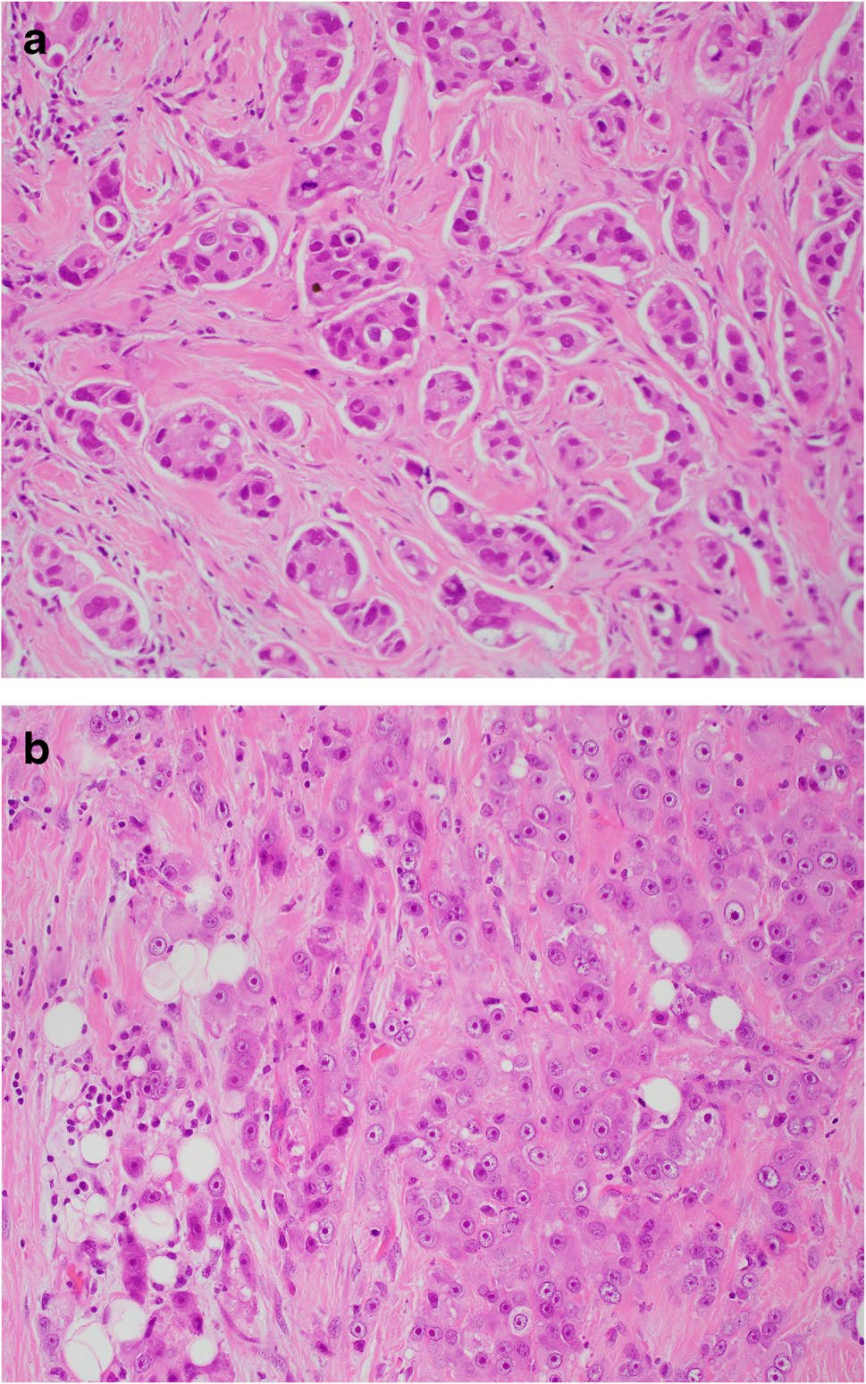
Examples of invasive carcinoma with apocrine differentiation (**a**, **b**)

**Fig. 11:**
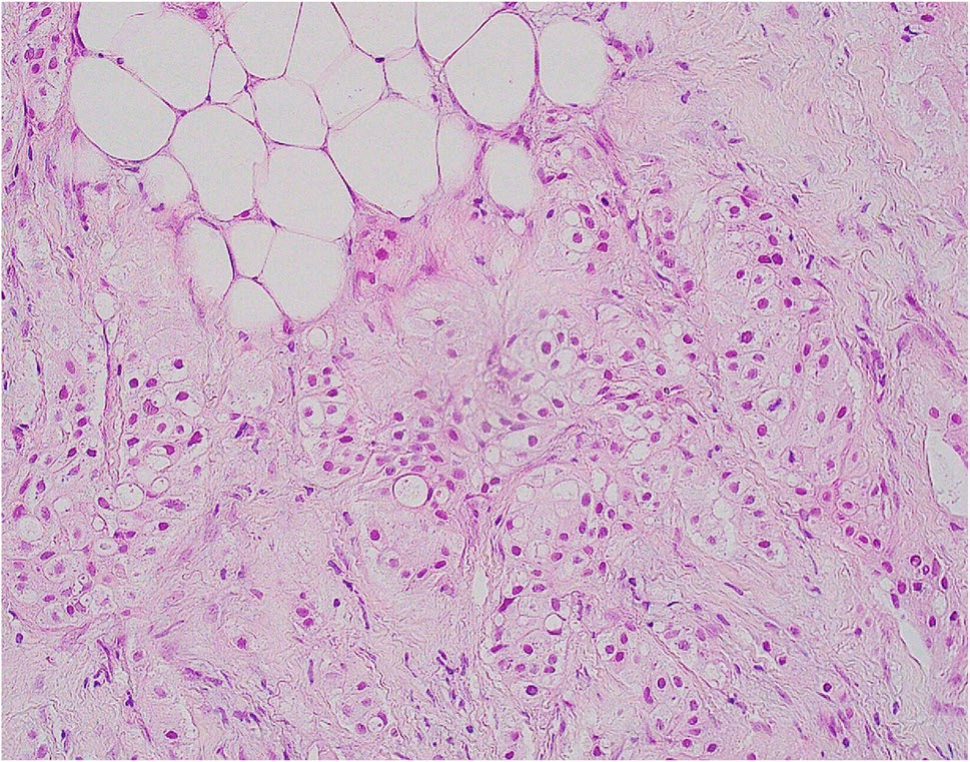
Invasive carcinoma with apocrine morphology composed of cells with foamy cytoplasm resembling histiocytes (histiocytoid carcinoma)

The 2019 edition of the WHO Classification of Breast Tumours utilises the term ‘carcinoma with apocrine differentiation’ to describe tumours with specific morphology characterised by ‘large cells with abundant eosinophilic granular cytoplasm and enlarged nuclei with prominent nucleoli, resembling apocrine sweat glands’ (Fig. 10) [41]. These are relatively rare tumours, tend to affect older women and are usually sporadic. Tumours present clinically, as a palpable, poorly defined, mass or may be detected on mammographic screening. Architecturally, most examples of carcinoma with apocrine differentiation resemble invasive breast carcinoma of no special type (NST), with a predominant solid growth pattern. Cytological atypia tends to be moderate or marked with intermediate or high mitotic activity such that these tumours are usually histological grade 2 or 3. ADCIS may co-exist, with intermediate or high nuclear grade features. Apocrine morphology may also be seen in other invasive subtypes including invasive micropapillary, mucinous and pleomorphic invasive lobular carcinoma (ILC) [42]. Rare carcinomas composed of apocrine cells (GCDFP-15 positive) with foamy granular cytoplasm resembling histiocytes have been described (Fig. 11) [43, 44]. These tumours, often referred to as histiocytoid carcinomas, may show ductal or lobular type morphology, with loss of E-cadherin expression in some examples, and do not represent a special subtype of invasive carcinoma [45–47].

**Fig. 12:**
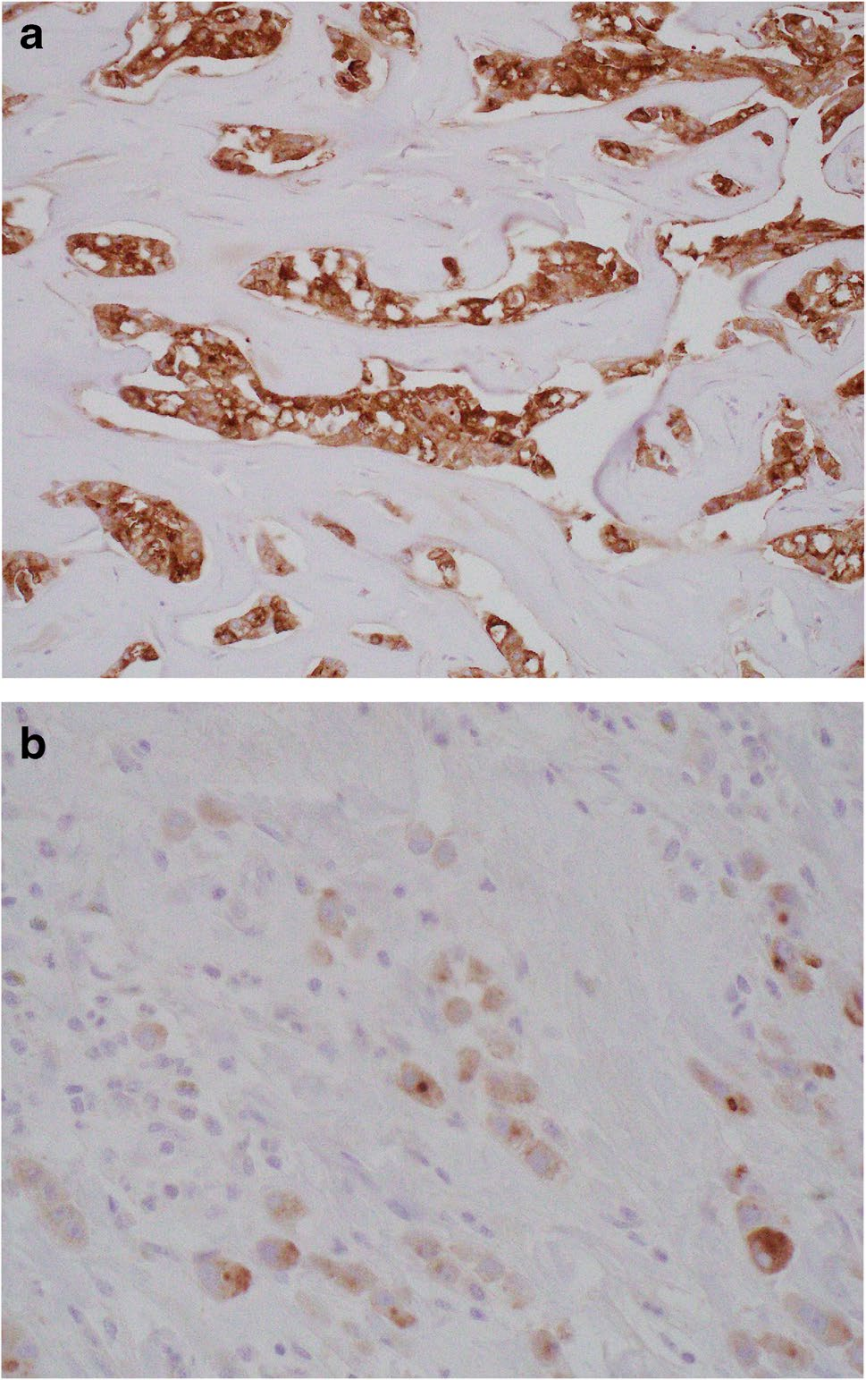
Gross cystic disease fluid protein-15 (GCDFP-15) expression, demonstrated on immunohistochemistry, in carcinoma with apocrine differentiation (**a**). GCDFP-15 expression in invasive lobular carcinoma with apocrine morphology (**b**)

Regardless of histological subtype, carcinomas with apocrine differentiation are associated with cellular expression of GCDFP-15 (Fig. 12) [48, 49], GATA-3 expression [50] and AR positivity [50–52]. GCDFP-15 expression may be reduced or lost in higher stage tumours [53]. Although typically hormone receptor negative, tumours fulfilling the morphological criteria for a diagnosis of ‘carcinoma with apocrine differentiation’ may show varying degrees of ER and PR positivity [51, 54].

There is little available information on the likely origin of apocrine carcinoma. Similar genetic alterations identified in papillary apocrine change and adjacent apocrine carcinoma, DCIS and invasive, suggest a possible pathogenetic link between apocrine hyperplasia and carcinoma [55]. Similarly, early reports of increased HER-2 and C-myc expression in benign and atypical apocrine lesions may indicate early oncogenic events in the development of apocrine malignancy [56, 57].

The differential diagnosis of invasive apocrine carcinoma includes apocrine and atypical apocrine adenosis, as discussed above, granular cell tumour, carcinoma with an oncocytic pattern and histiocytic proliferations [41]. Granular cell tumours are characterised by dense cytoplasmic eosinophilia and are ER negative. There is no nuclear atypia. Granular cell tumours do not express CKs and are strongly CD68 and S100 positive. Carcinoma with an oncocytic pattern is rare and may express GCDFP-15 and HER-2 but is usually ER positive [58]. Histiocytoid carcinoma may be confused with histiocytic inflammation. Awareness of the potential pitfall and judicious use of IHC will clarify the diagnosis.

**Fig. 13:**
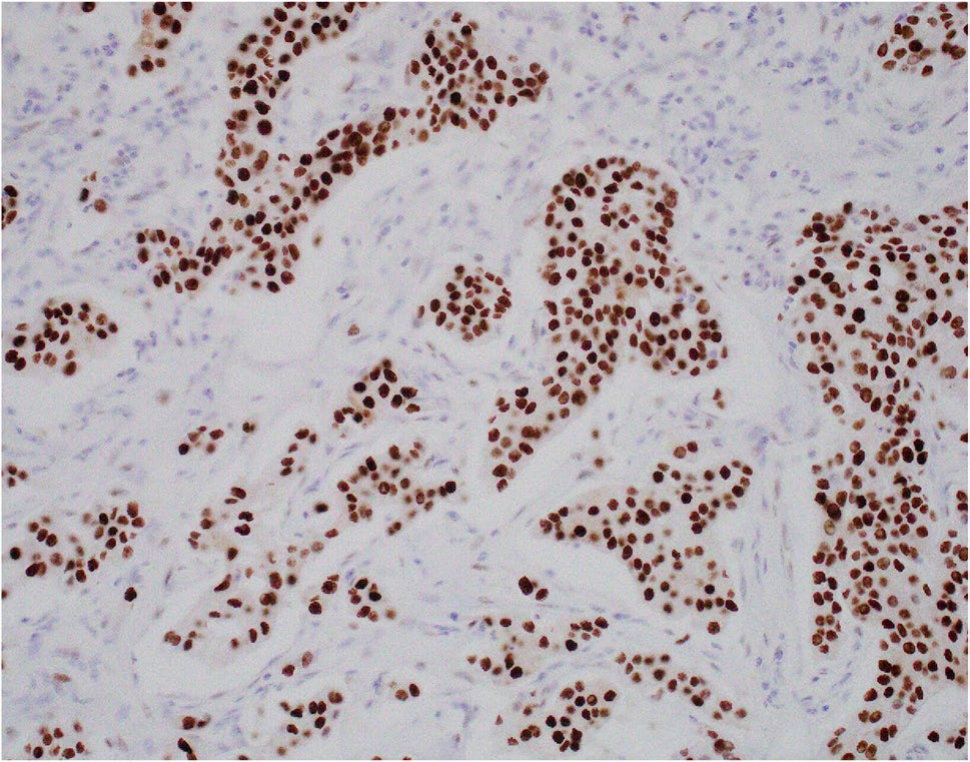
Androgen receptor positivity, demonstrated on immunohistochemistry, in carcinoma with apocrine differentiation

**Fig. 14:**
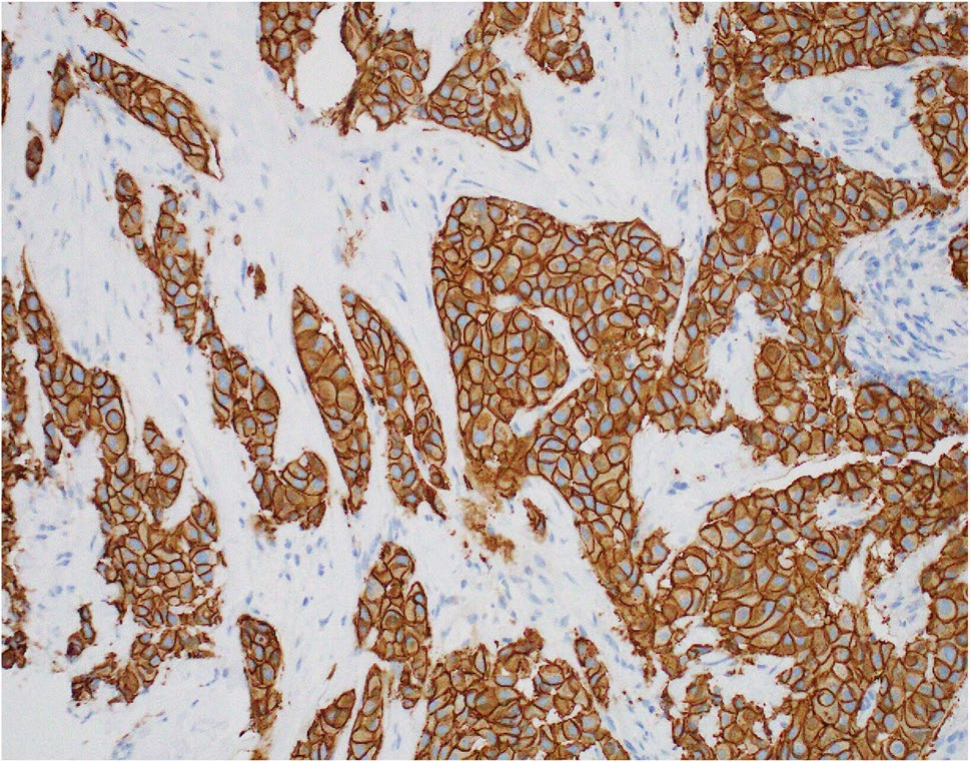
Carcinoma with apocrine differentiation showing HER-2 positivity, demonstrated on immunohistochemistry

The term ‘pure apocrine carcinoma’ has been used to define tumours characterised by ER and PR receptor negative status, AR positivity in at least 10% of tumour cell nuclei on IHC (Fig. 13) and classical apocrine morphology in at least 90% of the tumour [51]. Pure apocrine carcinoma is rare, accounting for less than 1% of all breast tumours. Applying these strict criteria, Vranic et al. [59] demonstrated that pure apocrine carcinoma will categorise as either HER-2positive (Fig. 14) (57%) or as triple negative on IHC. The HER-2 positive subset of pure apocrine carcinoma shows transcriptional overlap with the molecular signature of the so called [12] molecular apocrine tumours (MATs) [51, 60] and the triple negative subset with a cohort of triple negative breast cancers (TNBCs) known as luminal androgen receptor (LAR) tumours [61], discussed below.

The term ‘molecular apocrine tumour’ (MAT) was introduced in 2005 by Farmer et al. [60] who classified 49 breast tumour samples, using principal components analysis and hierarchical clustering, into luminal, basal and molecular apocrine subgroups. The MAT subgroup comprised six tumours with apocrine morphology, hormone receptor negative status, expression of luminal CKs and high rates of AR signalling at transcriptional level. HER-2 amplification was identified in three of the six MATs suggesting a possible link between HER-2 signalling and the molecular apocrine phenotype. In a subsequent genome-wide transcript expression analysis of 99 primary breast cancer samples and eight cell lines, Doane et al. identified ten (of 41) hormone receptor negative tumours with paradoxical expression of AR and multiple other genes, known to be ER targets or typically expressed in ER positive tumours [62]. Strong apocrine morphology was observed in seven of the 10 tumours.

Recent studies focusing on the heterogeneity of TNBC have identified four subtypes [63, 64], refined from an original six [26, 61], including a subset known as LAR tumours. These tumours, accounting for 10% of TNBCs, constitute a distinct subgroup with increased expression of AR mRNA and many gene expression features of ER positive luminal breast cancer [65]. LAR tumours show strong similarities with the MAT gene expression profile suggesting that this TNBC subgroup includes the HER-2 negative MATs [61].

Pure apocrine carcinoma, as defined by Vranic et al., includes a requirement that at least 90% of the tumour must display apocrine morphology in addition to an ER and PR negative and AR positive steroid receptor profile [59]. These tumours, therefore, constitute a subset of ‘carcinomas with apocrine differentiation’ as defined on the basis of morphology [41]. There is considerable overlap between pure apocrine carcinoma and MATs but these tumours are not entirely equivalent. In a series of 58 MATs defined, using quantitative reverse transcription PCR, as ER negative and AR, FOXA1and AR related genes mRNA positive, almost all tumours were ER and PR negative on IHC [66]. However, only 58% showed AR expression on IHC and apocrine morphology was observed in only 4 tumours (7%). HER-2 IHC positivity was observed in 67% and GCDFP-15 IHC positivity in 57% of tumours. One or other marker was expressed in 97% of tumours and these authors proposed a composite molecular and IHC signature for recognition of MAT.

Recognition of pure apocrine carcinomas and MATs in clinical practice is likely to become more important as our knowledge of tumour biology advances and new treatment strategies emerge [67]. Interaction between androgen and the HER-2 signalling pathway in hormone receptor negative tumours has been demonstrated [68, 69] with impaired cell growth following AR blockade [68]. Reported rates of AR positivity in TNBC range from 0 to 53% [70], likely a reflection of different methodologies used to assess AR and of uncertain prognostic significance [71–73]. Early clinical studies of anti-androgen therapy in patients with advanced AR-dependent, ER-independent invasive breast carcinoma have reported encouraging results [74–76]. Carcinomas with apocrine differentiation frequently harbour mutations of *TP*53 and PIK3CA/PTEN/AKT genes [59, 77, 78]. The presence of PIK3CA mutations, particularly in the TNBC variants, offers potential for cyclin-dependent kinases 4 and 6 (CDK4/6) inhibitor therapy [77, 79, 80]. Asghar et al. have recently demonstrated that the LAR subset of TNBC proved highly sensitive to CDK4/6 inhibition both in vivo and in vitro [81].

Current WHO recommendations advocate the use of the term ‘carcinoma with apocrine differentiation’ for tumours that display apocrine features on morphology alone with determination of ER, PR and HER-2 status as for all invasive breast carcinomas [41]. The combination of apocrine morphology, hormone receptor negative status and AR expression in a subset of HER-2 positive tumours and TNBCs identifies some but not all MATs [82]. Documentation of tumour AR status using IHC, while not yet standard of care, may become part of the clinical work-up of carcinomas with apocrine differentiation [83].

### Prognosis of apocrine carcinoma

Data on the prognosis of apocrine carcinoma are conflicting and difficult to interpret due to the use of varying definitions. Applying the definition of Vranic et al. [59], pure apocrine carcinoma appears to have a worse disease-free survival rate than invasive breast carcinoma, NST [52]. In the study of patients with transcriptionally defined MATs, Lehman-Che et al. reported decreased 5-year disease free and overall survival rates compared with patients with basal-like tumours [66]. Lehmann et al. observed a decreased relapse-free survival time in patients diagnosed with LAR tumours compared to other TNBCs [61]. Other studies have reported improved overall and disease-free survival in patients with AR positive TNBC compared with other TNBCs [84, 85]. Data on response to neoadjuvant therapy are scarce and also conflicting [63, 86].

## Apocrine lesions on NCB

- Assessment of apocrine lesions on NCB can be particularly challenging. Careful attention to nuclear detail and architectural configuration is important to avoid both over and under-diagnosis.
- Apocrine metaplasia in benign cysts may show complex papillary hyperplasia which should be distinguished from the architectural patterns of DCIS. Considerable variation in nuclear size is also commonly observed in benign apocrine change but should be less than threefold. The presence of necrosis is abnormal and should raise suspicion of an atypical or malignant process even if other features are not well developed.
- The distinction of apocrine adenosis and atypical apocrine adenosis is based on assessment of variation in nuclear size. Definitive categorisation may be difficult on NCB depending on the extent of abnormality present in the biopsy material. A decision to observe or excise should be made at multidisciplinary team review. Both lesions may mimic invasive carcinoma. Appreciation of the lobular architecture and appropriate use of IHC will assist diagnosis.
- The distinction of AAH from non-high grade ADCIS is difficult even on excision specimens, partly due to lack of firm diagnostic criteria. Examination of additional levels may assist the diagnosis. In the absence of obvious features of malignancy, e.g. cytonuclear atypia, necrosis, atypical mitotic activity or if the lesion is limited in extent (< 2 mm) and without significant peri-ductal fibrosis and peri-ductal lymphocytic infiltrate, a B3 or B4 designation is advised with a recommendation for multidisciplinary review and further sampling to fully evaluate the abnormality. In view of the scarcity of upgrade rate data for atypical apocrine proliferations, surgical excision may be preferable to vacuum assisted excision.
- The differential diagnosis of carcinoma with apocrine differentiation is discussed above. In particular, consideration of granular cell tumour and histiocytic inflammation on NCB material will prevent over diagnosis of malignancy.

## Abbreviations

AAH: Atypical apocrine hyperplasia
ADCIS: Apocrine ductal carcinoma in situ
ADH: Atypical ductal hyperplasia
AR: Androgen receptor
CK(s): Cytokeratin(s)
DCIS: Ductal carcinoma in situ
ER: Oestrogen receptor
HER-2: Human epidermal growth factor receptor 2
IHC: Immunohistochemistry
LAR: Luminal androgen receptor
LCIS: Lobular carcinoma in situ
MAT: Molecular apocrine tumour
NCB: Needle core biopsy
NST: No special type
pLCIS: Pleomorphic lobular carcinoma in situ
PR: Progesterone receptor
